# Experiences of general practitioners in the Ga-Rankuwa and Mabopane areas in dealing with patients who have sexual problems

**DOI:** 10.4102/phcfm.v7i1.878

**Published:** 2015-12-09

**Authors:** Benjamin Mills, Indiran Govender, Jannie Hugo

**Affiliations:** 1 Department of Family Medicine, Sefako Makgatho Health Sciences University, South Africa; 2 Department of Family Medicine, University of Pretoria, South Africa

## Abstract

**Background:**

Sexual problems are common. Many patients with sexual health dysfunction use self-help literature or are often managed in general practice. However, many general practitioners (GPs) find it difficult to discuss sexual health issues because they feel uncomfortable with this and lack training in these matters. These GPs are now referring patients with sexual dysfunction to specialists.

**Aim:**

We sought to explore how GPs working in the Mabopane and Ga-Rankuwa areas of handle sexual problems of their patients.

**Setting:**

The setting was the Mabopane and Ga-Rankuwa areas of North-West Tshwane, in Gauteng Province.

**Methods:**

A qualitative study comprising eight free attitude interviews with purposefully selected four male and four female GPs. All interviews were conducted in English and tape-recorded. Field notes in the form of a detailed diary was kept. The tapes were transcribed verbatim, and the transcriptions were checked against the tapes for omissions and inaccuracies.

**Results:**

Six themes emerged from the interviews: causes of sexual problems; presentation of sexual problems to the doctor; management of sexual health problems; sex is a taboo topic; society's need for sexual health discussions, and these discussions have already begun; previous limited exposure and training, and a need for more sexual health training.

**Conclusion:**

This study confirms earlier findings that patients could be either reluctant to discuss their problems or are open about them when presenting to doctors with sexual dysfunction. GPs were not exposed to sexual health training at medical school and, because of this shortcoming, felt that training in sexual medicine should be part of the curriculum.

## Abstrait

**Expériences des médecins généralistes dans les districts de Ga-Rankuwa et de Mabopane dans leurs rapports avec les patients qui ont des problèmes sexuels**.

**Contexte:**

Les problèmes sexuels sont courants. Beaucoup de patients souffrant de troubles sexuels utilisent des documents d'auto-thérapie ou sont souvent traités chez des généralistes. Cependant, de nombreux médecins généralistes (MG) ont du mal à discuter des problèmes de santé sexuelle car ils ne se sentent pas à l'aise pour parler de cela et n'ont pas eu de formation dans ce domaine. Ces MG envoient maintenant les patients ayant des troubles sexuels à des spécialistes.

**Objectif:**

Nous avons essayé de déterminer comment les MG des districts de Mabopane et de Ga-Rankuwa gèrent les problèmes sexuels de leurs patients.

**Lieu:**

Les districts de Mabopane et de Ga-Rankuwa au Nord-Ouest de Tshwane, dans la province de Gauteng.

**Méthodes:**

Une étude qualitative comprenant huit entretiens libres avec quatre hommes et quatre femmes médecins délibérément choisis. Tous les entretiens se sont déroulés en anglais et ont été enregistrés. Des notes ont été prises sur le terrain sous forme de journal détaillé. Les bandes enregistrées ont été transcrites textuellement, et les transcriptions ont été contrôlées avec les bandes pour relever les omissions et les erreurs.

**Résultats:**

Six thèmes sont apparus dans ces entretiens : les causes des troubles sexuels ; la présentation des problèmes sexuels au médecin; la gestion des problèmes de santé sexuelle; le sexe est un sujet tabou; la nécessité d'avoir des discussions sur la santé sexuelle dans la société et, ces discussions ont déjà commencé; exposition et formation préalables limitées, et nécessité d'avoir plus de formation en matière de santé sexuelle.

**Conclusion:**

Cette étude confirme les résultats précédents que les patients sont soit réticents de discuter de leurs problèmes, soit en parlent librement quand ils vont chez le médecin avec des troubles sexuels. Les médecins n'ont pas eu de formation à la faculté de médecine en matière de santé sexuelle et, en raison de cette lacune, ils pensent que la médecine sexuelle devrait faire partie du programme de formation.

## Introduction

In this article human sexuality refers to the integration of somatic, emotional, intellectual and social aspects of sexual being in ways that are positively enriching and that enhance personality, communication and love.^1^

Sexual problems are common at any age and in any race. The most common problems are loss of sexual drive, anorgasmia, vaginismus in women and erectile failure and premature ejaculation in men. Approximately 10% of medical outpatients experience sexual dysfunction, and this percentage is increasing.^2^ In a gynaecological clinic, up to 38% of women report anxiety and inhibition during sexual activity, 16% complain of lack of pleasure and 15% have difficulties reaching orgasm.^3^ Up to 40% of middle-aged men report some kind of sexual dysfunction.^4^ The dysfunction may be purely psychological or physical, but is usually a combination of the two.^5^ Once established, and regardless of the source, any sexual disorder is likely to induce a vicious cycle that generates anxiety, guilt, shame and fear – all of which in turn make sex even less satisfactory.

Many patients use self-help literature or are often managed in general practice.^6^ An increasing number of patients are referred to urologists, gynaecologists, psychiatrists and psychologists. Specialty psychosexual services have arisen to manage sexual dysfunction.^7^ This separation and specialisation sometimes deprives patients of a holistic approach to their difficulties.

Many people believe that their general practitioner (GP) is a suitable professional to whom they can confide their sexual difficulties. Attitudes towards sexuality are changing, and people expect their GP to be able to ask them about sexual problems. However, many GPs find it difficult to discuss sexual health issues because they feel uncomfortable with this and lack training in these matters.^8^

Many physical disorders and surgical procedures may lead to sexual problems.^9^ Consequently, a thorough medical history and physical examination by the GP is mandatory.^2,9^

GPs have observed that, in spite of the drastic changes in public attitudes towards sexuality over recent years, many people still find it embarrassing to talk honestly about sexuality. Some sexual problems are covertly presented in the form of either emotional or psychological symptoms (such as depression or request for a change of contraceptive pill, or reporting of a vaginal discharge). There are some sexual problems that can be presented directly to the GP, such as sexually transmitted infections and fear of HIV infection.^9^

GPs report that details of the sexual values, ideas and behaviour of the client are important, but useful only if sought in a non-judgemental fashion. A thorough description of the sexual difficulty and interpersonal circumstances surrounding it is essential. The specific language chosen by the doctor to discuss the problem should reflect the patient's level of understanding. It is sometimes beneficial to reassure the reticent, anxious patient of the confidentiality of doctor-patient communication. Preferences as to activities and partners should be explored, including both homosexual and heterosexual experiences.^2^

Referral is indicated when severe underlying psychopathology is suspected, marital discord is very serious, the patient is unwilling, or unable, to cooperate, or couple-directive sexual therapy undertaken by the GP has failed.^2^ Educational sessions are best conducted with both partners present because this improves mutual understanding and communication.^1^

In some countries there are institutions for research and teaching in sexology within the universities, which GPs can attend.^1^ Britain's Royal Colleges view sexual medicine as an important part of training, but there is little consistency about the quality or duration of such training.^10^ In South Africa there has been no substantial change in the training of GPs, although a World Health Organization report in 1975 emphasised the need for health professionals to be trained in human sexuality.^1^

In order to develop a better understanding of problems of human sexuality it is necessary for GPs to develop healthy attitudes to sexuality, marriage and contraception. In turn, he or she can transmit this assurance to those clients who seek help for what they consider to be abnormal behaviour in themselves or their partners.^1^

In order to approach the topic with confidence, the GPs must have accurate scientific knowledge regarding the facts of human reproduction and human sexuality; they must know what common sexual problems are and how to deal with them, and must know when the solution to a problem is beyond their ability and requires referral to a specialist.^1^ To enhance their ability to help people with sexuality problems it is essential for GPs to develop their skills in communication.^1^

Other health professionals, besides GPs, that need to be educated and trained in this arena include undergraduate and postgraduate medical students, social workers, psychologists, teachers and marriage counsellors, because they come into contact with individuals with sexual health issues. An interdisciplinary approach is needed for training the GP to include psychology, psychiatry, gynaecology, urology, paediatrics, nursing, social work and health education.^1^ Human sexuality should be developed as an autonomous discipline in the training of health professionals and become a recognised component of general health services, particularly family health.^1^

The aim of this study was to find out how GPs working in the Mabopane and Ga-Rankuwa areas of Gauteng Province, South Africa, handle sexual problems in their patients.

## Research methods and design

### Study design

A qualitative study using free attitude technique interviews.

### Setting

The study was conducted in Ga-Rankuwa and Mabopane, two large black townships, 32 km north-west of Pretoria, South Africa. These two areas were previously part of the former Bophuthatswana and mainly Tswana speaking. Amongst these Tswana people could be found the Sotho, Shangaan, Nguni (Xhosa and Zulu) and Venda people.

### Study population and sampling strategy

All 45 private GPs working in the Ga-Rankuwa and Mabopane areas constituted the population. Purposeful sampling was carried out, and eight free attitude interviews with four male and four female GPs were conducted. Purposeful sampling technique was used because the logic and power of purposeful sampling lies in selecting information-rich cases for study in depth.

All the private general practitioners (GPs) working in the Ga-Rankuwa and Mabopane areas were contacted telephonically. Three were not willing to participate in the study. The researcher interviewed doctors until data saturation was reached. The criteria for enrolment were:

- equal number of males and females;
- equal number of doctors with less than, or more than, 8 years’ experience.

### Data collection and analysis

The free-attitude technique was used. The technique develops the crucial attitude that Smaling refers to as ‘open mindedness's’.^11^ The free attitude technique was used for all the eight participants.

The interview began with the researcher giving some short information about himself and the frame of reference of the interview. The exploratory question was the only question asked which was formulated in an open-ended way so that it did not contain any suggestion. The researcher facilitated the interview by using the techniques of summarisation, reflection and clarification. The exploratory question used in the interviews was: ‘How do you handle sexual health problems amongst your patients?’ Each interview took between 30 to 45 min. The interviews, which were conducted by BM, were conducted in the practice of the GPs. All of the interviews were held in English.

All of the interviews were tape-recorded. Field notes in the form of a detailed diary were taken by the researchers. These were discussed daily with the other researchers. The tapes were transcribed verbatim, and the transcriptions were checked against the tapes for any omissions and inaccuracies. Each interview took an average of eight hours to transcribe.

Three photocopies of each of the transcripts were made, and the master copy was kept untouched. Each transcript was reread several times by the researchers to ascertain what the interviewee said. The researchers read through all the field notes and interviews and made comments in the margins. Paper notes were attached containing the interviewers’ comments about how the data could be utilised. The cut and paste technique was used to finally organise the data into the themes that emerged from the interviews so that nothing was lost. The field notes of each interview were read together with the corresponding transcript to help the researchers to organise the data into themes and sub-themes.

### Trustworthiness

To aid in the trustworthiness and rigour of the research, the authors implemented various constructs as proposed by Lincoln and Guba. Although reliability and internal validity of qualitative research is often questioned, the use of trustworthiness is generally accepted as a means to remedy this issue.^12^ This construct consists of credibility, transferability, dependability and conformability.

Credibility, also known as internal validity in quantitative research, refers to a study measuring what it has intended to measure.^13^ For this study, the authors implemented triangulation in the process of data collection. Triangulation from two sites in Tshwane, Mabopane and Ga-Rankuwa was implemented. According to Shenton,^12^ when similar results are observed from different sites the credibility of the study is increased. Also, to improve trustworthiness and triangulation of the observations purposeful sampling from an equal number of male and female GPs, and an equal number of doctors with more or less than 8 years’ experience was conducted. Here the authors sought to improve credibility by having a range of demographic characteristics of participants to assist in the representativeness of the sample. Another key aspect of the credibility of this study was an intention to improve the honesty of the information provided by the participants. In order to address this, participants were allowed opportunities to withdraw from the study. Participants were informed verbally and in writing (in the informed consent document) about the research and that they could withdraw from the study at any point in time.

The authors also attempted to improve credibility by having regular discussions and debriefing sessions with the interviewer and the other researchers. The interviewer also underwent the same interview process with fellow researchers to identify flaws and potential interviewer bias. These interviews amongst fellow researchers were used to clarify interview questions, prompts and individual biases. The authors referred to this as self-clarification. The authors hoped that conducting interviews with the researchers before the interviews would sensitise the authors to possible personal bias and opinions. These could then be reduced.

Nomsa Malete (NM), previously employed by Medunsa as a research and teaching assistant, was an external expert who verified the transcription, themes, and analysis of the data. The external expert researcher checked the analysis and confirmed the accuracy of the information of the transcripts and themes obtained. The themes subsequently identified were discussed with the participants (this provided respondent validation and/or member checks), NM and co-researchers. Reflexivity, which refers to the researcher's awareness of the self as a research instrument,^13^ was achieved by the researchers conducting free attitude interviews on one another to minimise, or at least become aware of, their own prejudices, viewpoints or assumptions regarding the phenomena under investigation.^13^ Free attitude interviewing cannot always provide accurate responses on sensitive issues. To reduce the potential of this occurring, it was hoped that in the individual interview method (as opposed to a group discussion) participants may have felt more comfortable to discuss sensitive issues where privacy, anonymity and confidentiality may be assured. Field notes in the form of a research diary and the audio tapes, which were transcribed verbatim, were used to verify the information recorded.

Confirmability, which describes the objectivity of the study,^12,13^ was addressed by providing detailed information regarding the research method used in the selection of participants, questions posed in the interviews and reasons for this research methodology. This enables other researchers to make informed decisions about this study. The study also provides a basis on which other researchers can replicate it. Along with this, the researchers have provided verbatim quotes from the participants’ narratives to indicate that these were not altered in any way.

## Ethical considerations

Informed written consent was obtained from all the participants, and they were assured of confidentiality and anonymity. Code names were used from the transcription phase onwards to make recognition of individual participants impossible. Ethical clearance was obtained from Medunsa Research and Ethics Committee.

## Results

The characteristic profile of the eight participants are summarised in Table 1. The age group of the participating GPs ranged from 30 to 47 years.

**TABLE 1:** Profile of participants.

Participant number	Age (yrs)	Sex	Years of practice	Feels that patients are reluctant to discuss sexual problems	Personally feels that sex is a taboo subject	Never exposed to training for sexual problems	Feels there is a need fortraining
1	35	M	> 8	Yes	N/S*	Yes	Yes
2	37	F	> 8	N/S	Yes	Yes	Yes
3	42	F	> 8	Yes	Yes	N/S	N/S
4	41	F	> 8	Yes	N/S	N/S	Yes
5	36	M	> 8	N/S	Yes	Yes	Yes
6	30	M	> 8	Yes	Yes	N/S	Yes
7	47	M	> 8	N/S	No	Yes	Yes
8	46	F	> 8	N/S	N/S	N/S	N/S

*, N/S, Not mentioned.

### Themes

There were six themes that emerged from the transcripts as follows: causes of sexual problems; presentation of sexual problems to the doctor; management of sexual health problems; sex is a taboo topic; society has a need for sexual health discussions, and these discussions have already begun; previous limited exposure and training, and a need for more sexual health training.

### The causes of sexual problems

All doctors, except one, stated that organic or non-organic problems could cause sexual problems. The doctors mentioned sexually transmitted diseases, diabetes mellitus and some drugs can, as a side effect, result in sexual problems. Non-organic problems were related to psychological conditions. The participants stated that sexual problems affect almost everyone in one way or another. The need to take a good history to determine the cause of the sexual problem was emphasised. Quotes from participants to support this theme are:

‘Psychological or organic problems cause sexual problems.’ (NM, female, 42 years old)

‘Find what could have led to the cause of the sexual dysfunction by going into the history, checking the treatment and checking for stress factors.’ (MM, female, 41 years old)

‘Nobody is exempted when it came to sexual problems.’ (PP, male, 30 years old)

‘Sexual problems could emanate from psychological problems and organic problems like diabetes and side-effects of antihypertensive and antipsychotic drugs.’ (MG, female, 37 years old).

### Presentation of sexual problems to the doctor

All the doctors spoke about how patients with sexual problems presented to them, but there were variations in the way patients presented them depending on the demographic characteristics (e.g. gender, age) of the doctor, the patient and the actual sexual problem. Patients were more comfortable with older and same-sex doctors. Quotes from participants to support this theme are:

‘Patients were reluctant to talk about their sexual problems but sometimes a female would have difficulties relating the problems, but with a little bit of encouragement she may cry and then after she would pour out everything.’ (PM, male, 35 years old)

‘Male patients are reluctant to present to me because I am a woman doctor but I tell men to forget that I am a female doctor and just present the problem.’ (MM, female, 41 years old)

‘Females are more open to FEMALE doctors.’ (NM, female, 42 years old)

‘I see more male patients than females because there was that belief that male patients thought they were better understood as to what was happening if seen by a male doctor.’ (PM, male, 35 years old)

‘Patients are shy about sexual diseases and they use other terms. Older males present infection to me because they understood that I am a male. Older females do not open to me because I am a young male doctor.’ (PP, male, 30 years old)

‘Some patients are frank about it whilst others are shy.’ (JM, female, 46 years old)

‘Some females present on behalf of their partners saying that they have no interest in sex anymore.’ (MM, female, 41 years old)

‘A lot of patients have sexual problems and they either present directly or in an indirect way.’ (MG, female, 37 years old)

### The management of sexual health problems of the participants

Almost all the participants (seven out of eight) expressed difficulties in approaching and managing sexual health problems. They found it easier to manage organic problems such as sexually transmitted diseases. They were uncomfortable in managing sexual problems of a psychological nature and commonly referred patients with these problems to specialists. To satisfy the patients and to ensure that they do not lose these patients from their practice, these doctors also gave placebo vitamin injections to the patients. Quotes from participants to support this theme are:

‘Sexual problems are not easy to handle.’ (PP, male, 30 years old)

‘Sexual problems were a big problem for me in the past but currently I sit and listen to patients and refer to books and journals when nothing seems to fit.’ (MM, female, 41 years old)

‘I give them injection or Vit B Co or refer them to specialists such as urologists.’ (PP, Male, 30 years old)

‘I do feel guilty about the way I manage these problems and all GPs are doing the same thing and I see this kind of practice as “survival of the fittest.”’ (PM, male, 35 years old)

‘I refer some patients to the psychologist if there is a stress element involved or to the urologist if there was a surgical problem.’ (JM, female, 46 years old)

‘I use diplomacy to appease patients as I do not know what is happening’ (TK, male 46 years old)

‘I manage patients by going into the sexual pattern or habits do basic examination and educate, before considering the need for investigation.’ (NM, female, 42 years old)

‘When nothing seems to fit, I give them an appointment date and go look up in books and journals. Also I refer these patients to urologist and psychologists and get feedback from these specialists.’ (MG, Female, 37 years old)

### Sex is a taboo topic

The participants felt that sex is not a topic patients or people in general would like to talk about openly. The African culture was stated as one of the reasons why people feel sex and sexual health is taboo. It was felt that parents do not talk to their children about sex, and people do not even talk openly about sex and sexual problems with their friends. Quotes from participants to support this theme are:

‘Sex is treated like a taboo among African people and these people don't talk sex just like that. And no, parents will not talk to a boy or a girl directly that you are not supposed to do that because of this and that, because it is treated like a hush subject.’ (MG, female, 37 years old)

‘Sex is treated like a taboo and there is no openness.’ (JM, female, 46 years old)

### Society has a need for sexual health discussions, and these discussions have already begun

The participants stated that the radio, sexual health talk shows on television and explicit publications on sex in magazines has made sex something nowadays to be openly talked about in a healthy way. Adolescent children are also discussing sex nowadays. This has put pressure on doctors to discuss and become comfortable with sexual health issues. Quotes from participants to support this theme are:

‘A lot of open-mindedness of the whole community actually from home to schools, to churches is seen these days.’ (PP, male, 30 years old)

‘There was a lack of communication as far as sex was concerned and that has led to other problems like sexually transmitted diseases, pregnancies and infertilities. If many people open up it would be easy for one to detect problems and be treated accordingly.’ (JM, female, 46 years old)

‘Sex talk shows on radio and TV and people like Dr Ruth and Dr Paul and many others have made sex something to be openly talked about in a more healthy way.’ (EK, male, 47 years old)

‘Sex in the past was confined to adults but now adolescents must be included in sex education because children as young as 13 and 14 are sexually active and it has to be accepted and they have questions of their own.’ (TK, male, 46 years old)

‘Patients have information through TV, magazines, newspapers, radio and internet.’ (MM, female, 41 years old).

### Need for more sexual health training

Participants stated that they were never exposed to sexual health training at medical school and therefore expressed a need for training in this field. They suggested that training could be in the form of literature or workshops. Training should be given at the undergraduate or postgraduate levels. Also, they mentioned that a family practice journal should include ‘the approach to such sexual health conditions’ (MG, female, 37 years old). They felt their feeling of inadequacy and low confidence in managing these problems stems largely from a lack of training. Quotes from participants to support this theme are:

‘In my clinical training, you could be told by trainers while in the wards that there was somebody with hepatomegaly or whatever but never would you be shown anybody with sexual problems.’ (PM, male, 35 years old)

‘I have no proper experience of how to handle sexual problems.’ (PP, male, 30 years old)

‘There was never a period where we were exposed to such situations, especially at medical school, and how to deal with such problems. The family practice curriculum and journal should include the approach to such conditions.’ (MG, female, 37 years old)

‘We are not really exposed at medical school and even outside.’ (TK, male, 46 years old)

‘It is a very difficult field we still need to know more about.’ (MM, female, 41 years old)

‘Any type of training will be needed either in the form of literature, workshops or undergraduate or postgraduate training.’ (EK, male, 47 years old)

‘I was not trained in counselling and stuff like that.’ (TK, male, 46 years old)

‘Listening, giving information and education could solve the problem but I lack the proper skills.’ (NM, female, 42 years old)

‘I am puzzled why the medical institutions did not look at the issue of sexual health problems.’ (JM, female, 46 years old)

### Integration of themes

**FIGURE 1:**
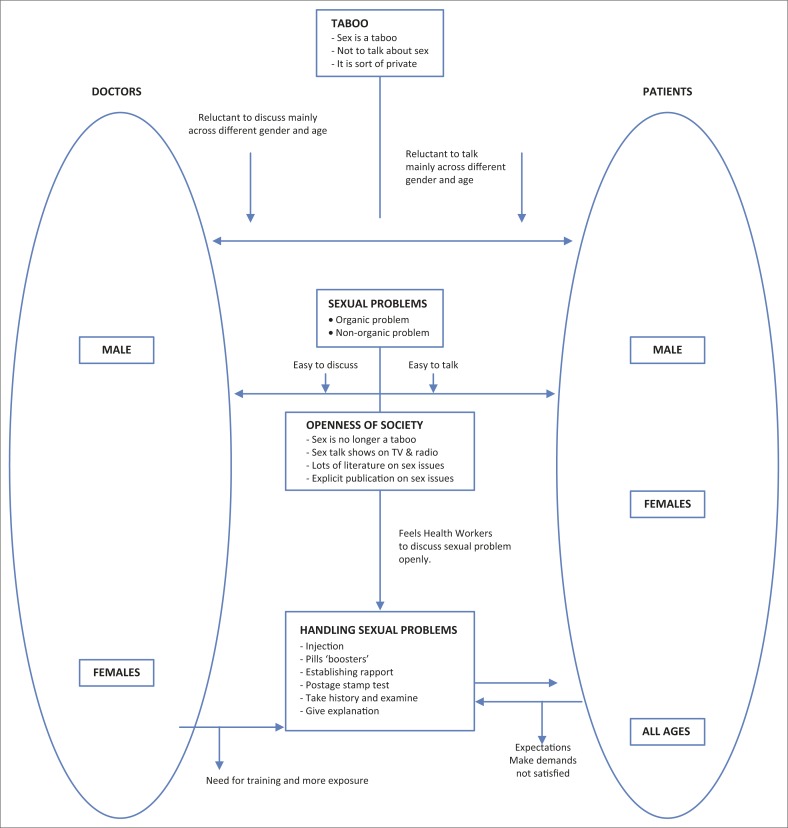
Integration of the themes between the general practitioners and their patients that emerged from the data.

The way in which the various themes can be integrated and related to each other is shown diagrammatically in Fig. 1.

The doctors perceived that patients were reluctant to consult them about their sexual problems. Most of the doctors stated that they were not exposed to sexual health problems at medical school and felt a need for more information and training in this area. Some of the doctors still felt that sex is a taboo topic.

## Discussion

The results indicate that doctors did not manage their patients with sexual problems according to evidence-based guidelines. In the case of non-organic problems, the GPs prescribed vitamin tablets, vitamin B complex injections and hormones. However, vitamins are not recommended, and one would seldom prescribe hormones because giving testosterone is contra-indicated in prostatic carcinoma, which may be undetected. Assessment of serum testosterone may be a useful investigation before prescribing testosterone,^14^ but none of the doctors mentioned this test. In Portugal, too, most GPs do not consult the guidelines about diagnosis and treatment of sexual dysfunction.^15^

About 90% of sexual health problems are psychological in origin, but it is essential not to overlook medical problems such as diabetes mellitus.^2,16^ In the doctors’ management of sexual problems with psychological causes, no mention was made about excluding diabetes mellitus.

Explicit sexual education, which is an important first step in managing sexual disorders, is not being undertaken by the doctors. Directive sexual therapy was not cited, which is indicated if a psychological basis for the dysfunction is discovered, or if adequate treatment of an underlying medical condition fails to fully alleviate the difficulty. No mention of directive sex therapy was made by the GPs as one of the ways to manage non-organic sexual problems.

No doctor said anything about making use of the ‘squeeze’ technique in patients with premature ejaculation. Although this technique is controversial it is still widely used by experts in the field.^17,18^ The treatment of vaginismus often includes the use of graduated dilators, but none of the doctors mentioned this. The doctors also did not state how they treated orgasmic dysfunction, where particular emphasis is placed upon instructing the woman in techniques of self-stimulation. In dyspareunia, the stage of intromission is gradually negotiated in many small steps and the couple is instructed to use copious amounts of artificial lubricant, but nothing was said by the GPs with regard to this type of treatment. Doctors said sexual problems could be caused by smoking and the use of alcohol, but they never stated that they would ask these patients to stop smoking and taking alcohol.

Most of the patients with non-organic sexual problems were referred by the GPs to gynaecologists, psychiatrists, urologists or psychologists. However, the primary care physician should be able to handle most of non-organic sexual problems.^2^ Referral is indicated when: (1) severe underlying psychopathology is suspected; (2) marital discord is very serious; (3) directive sexual therapy has been undertaken by the primary care physician.^2^ Nobody mentioned the use of Viagra, which is effective in patients with erectile dysfunction due to diabetes mellitus or atherosclerosis, and after radical prostatectomy and spinal cord injury.^15^

It could be argued that the way in which these GPs managed sexual problems was affected by their thinking about sexual issues. The doctors who felt that sex is a taboo topic were reluctant to enquire into patients’ sexual values, ideas and behaviour, which are very important.^2^ There is strong evidence that GPs do not discuss sexually related issues as often as patients would like. One important enquiry that GPs need to make is to ascertain what actually happens (and what does not happen) during sexual activity. Most GPs do not address sexual health issues proactively with patients, and this needs attention.^19^

The doctors who felt that discussions about sex are out in the open could discuss sexual issues with the patients. Research has shown that taking a detailed sexual history and making a risk assessment are key skills for making a diagnosis and care plan. There are sensitive questions that the GP must ask so as to obtain a correct sexual history and determine the further management of the patient.^20^

Most of the doctors said they were never exposed to sexual problems at medical school and this could explain their difficulty in handling sexual problems. They felt they needed training in sexual problems to help them handle sexual problems in their patients. Lack of training, knowledge or education of GPs about sexual dysfunction has been reported to be the most important barrier faced by them when dealing with sexual dysfunction.^21^ GPs in other countries have also stated that their training for their medical degree was inadequate when it comes to sexual health problems amongst patients.^22^ Besides the United Kingdom and Portugal, research in Australia has also pointed out the need for developing, improving and evaluating strategies to improve the management of sexual health problems by GPs.^6^ Some countries in Europe have identified sexual health as a priority public health area and, given the need indicated by the GPs in this study, and the country's national HIV and/or AIDS epidemic, South Africa also needs to follow this model.^23^

## Limitations

All participants were black GPs working in Ga-Rankuwa and Mabopane, hence these results may not reflect findings of GPs with other race groups.

The researchers aimed, not for generalisability, but for transferability through a rich description of the experiences of GPs of patients with sexual health problems in the context of urban black townships.

## Recommendations

Since doctors were not able to manage problems because of their lack of training at medical school, it is recommended that sexual medicine should become part of the curriculum at medical school. Firstly, this should include how to take a sexual history and, secondly, once a sexual problem is uncovered how to manage it. This study could be used as a pilot for a larger multicentre study.

## Conclusion

The eight GPs in this study did not manage sexual problems according to recommendations in the literature. This study confirms earlier findings that patients could be either reluctant or open to discussing sexual problems when presenting to doctors. The reason for this could be either cultural or the openness of current society, as indicated by the GPs. These GPs were not exposed to sexual health training at medical school and, because of this shortcoming, the GPs felt that there is a need for training in sexual medicine, which should be part of the curriculum at medical school.
